# Label-Free and Ultrasensitive APE1 Detection Based on Hybridization Chain Reaction Combined with G-Quadruplex

**DOI:** 10.3390/biom15091275

**Published:** 2025-09-03

**Authors:** Yarong Zhang, Hongyan Ma, Zhenyao Gao, Miao Li, Fan Yang, Lingbo Sun, Yuecheng Zhang

**Affiliations:** 1 Key Laboratory of Analytical Technology and Detection of Yan’an, College of Chemistry and Chemical Engineering, Yan’an University, Yan’an 716000, China; 2 Medical College of Yan’an University, Yan’an University, Yan’an 716000, China

**Keywords:** apurinic/apyrimidinic endonuclease 1 (APE1), hybridization chain reaction (HCR), G-quadruplex, fluorescence

## Abstract

Apurinic/apyrimidinic endonuclease 1 (APE1) selectively cleaves the apurinic/apyrimidinic site (AP site) in DNA, playing a critical role in base excision repair and genomic stability maintenance. Aberrant APE1 expression has been linked to numerous diseases, including cardiovascular disorders, neurological conditions, and various cancers. However, existing methods for detecting trace levels of APE1 remain suboptimal for certain applications. To address this limitation, we developed an innovative biosensing platform for ultrasensitive APE1 detection by integrating APE1-specific site recognition with hybridization chain reaction (HCR)-based signal amplification, enabling enzyme- and label-free bioassays. In this system, APE1 recognizes and cleaves the AP site-containing hairpin (HP) probe, releasing a single-stranded HCR initiator that triggers cascaded HCR amplification. Owing to the high efficiency of HCR, this method achieves ultrahigh sensitivity, with a calculated detection limit of 1.0 × 10^−8^ U/mL. Furthermore, the biosensor demonstrates robust performance in cell lysates and is applicable for screening and evaluating APE1 inhibitors.

## 1. Introduction

Apurinic/apyrimidinic endonuclease 1 (APE1) is a multifunctional protein that plays a pivotal role in DNA repair and transcriptional regulation in humans [1,2]. As a core component of the base excision repair (BER) pathway, APE1 processes various forms of DNA damage, including single-strand breaks, alkylation, oxidation, and abasic sites, accounting for approximately 95% of the total apurinic/apyrimidinic (AP) endonuclease activity in human cells [3,4,5,6]. Beyond its canonical repair function, APE1 regulates gene expression through redox-dependent interactions with transcription factors linked to cancer, highlighting its dual role in maintaining genomic stability and modulating oncogenic signaling [7]. Dysregulation of APE1 expression has been implicated in the pathogenesis of multiple diseases, including cardiovascular disorders, neurological conditions, and various cancers [8]. Notably, APE1 is overexpressed in cervical, colorectal, and ovarian cancers [9]. Zhang et al. demonstrated that aberrant APE1 expression not only contributes to the oncogenesis of high-grade serous ovarian adenocarcinoma but also promotes tumor progression, invasion, and metastasis [10]. Given its critical involvement in disease mechanisms, APE1 has emerged as a promising diagnostic and prognostic biomarker, as well as a potential therapeutic target for cancer and other human diseases.

Conventional methods for APE1 detection, including polyacrylamide gel electrophoresis (PAGE) [11], enzyme-linked immunosorbent assay (ELISA) [12], high-performance liquid chromatography (HPLC) [13], and chemiluminescence assays [14], have been extensively employed in research and clinical diagnostics. Despite their widespread use, these techniques are hampered by significant limitations such as labor-intensive procedures, reliance on sophisticated instrumentation, and demanding sample preparation protocols. In response to these challenges, innovative sensing strategies have recently emerged. Kang et al. developed a rapid label-free biosensing platform utilizing molecularly gated hyperbranched rolling circle amplification, achieving a detection limit of 1.0 × 10^−4^ U/mL [15]. Similarly, Hu’s group designed a highly sensitive and selective fluorescent biosensor based on APE1 substrate structure engineering and TdT/Endo IV-assisted dual signal amplification [16]. These advanced methods represent substantial improvements in APE1 detection sensitivity, their performance in detecting trace levels of APE1 remains suboptimal for certain applications. Consequently, there remains an urgent need to develop novel APE1 detection methodologies featuring both ultra-high sensitivity and an extended linear dynamic range to meet the growing demands of clinical diagnostics and basic research.

The hybridization chain reaction (HCR) is an enzyme-free, catalytic DNA circuit that operates through toehold-mediated strand displacement [17]. Its mechanism involves two meta-stable DNA hairpin probes that remain dormant until an initiator strand triggers an alternating, cascade-like hybridization event. The initiator nucleates with the toehold domain of the first hairpin (H1), opening it to reveal a single-stranded region that then opens the second hairpin (H2). This process repeats, self-assembling into a long, nicked double-stranded nucleic acid polymer. Unlike conventional DNA amplification techniques, HCR offers distinct advantages, including enzyme-free operation and efficient isothermal amplification [18]. This process is primarily driven by an increase in system entropy rather than a reduction in overall free energy, resulting in rapid reaction kinetics. Due to its modular design, simplicity, and robust signal amplification capability, HCR has emerged as a powerful tool for diverse applications, ranging from molecular computing and nanomaterial assembly to intracellular imaging.

Thioflavin T (ThT) is a fluorescent probe initially developed for detecting misfolded proteins [19]. Structurally, it consists of benzothiazole and dimethylaniline moieties connected by a rotatable C-C bond. In solution, free rotation of these groups facilitates twisted intramolecular charge transfer (TICT) in the excited state, resulting in fluorescence quenching [20]. However, when ThT binds to rigid macromolecular structures, its rotational freedom is restricted, suppressing TICT and leading to a dramatic increase in fluorescence signal. Notably, ThT exhibits strong fluorescence enhancement upon interaction with DNA G-quadruplexes. Studies have demonstrated that ThT’s fluorescence intensity increases by up to 1700-fold in the presence of G-quadruplexes, compared to only ~250-fold for single- or double-stranded DNA [21]. This remarkable contrast underscores ThT’s high selectivity for G-quadruplexes over other DNA conformations, making it a valuable tool for G-quadruplexes detection.

In this work, we developed a novel fluorescent assay for ultrasensitive APE1 detection. By synergistically combining the cascade signal amplification of HCR with the strong fluorescence enhancement from ThT-G-quadruplex conjugation, this method achieves exceptional sensitivity, surpassing most existing APE1 assays by at least two orders of magnitude. Such superior performance enables precise determination of APE1 activity at the single-cell level, underscoring its potential for advanced biomedical research and clinical diagnostics.

## 2. Experimental

### 2.1. Cell Lines

Human breast cancer cell line MCF-7, lung cancer cell line A549, gastric cancer cell line AGS, embryonic kidney cells line 293T and gastric epithelium cell line GES-1 were obtained from the cell bank of Yan’an university medical experimental research center. MCF-7 cells were cultured in DMEM medium supplemented with 10% fetal bovine serum (FBS), 1% glutamine and 1% penicillin streptomycin (PEST). AGS, 293T and GES-1 cells were cultured in DMEM medium supplemented with 10% FBS and 1% PEST. A549 cells were cultured in RPMI-1640 medium supplemented with 10% FBS and 1% PEST. All the cell lines were cultured at 37 °C with 5% CO_2_ in 100% relative humidity.

### 2.2. Materials and Reagents

APE1 and 10× NEBuffer 4 (20 mM Tris-acetic acid, 50 mM potassium acetate, 10 mM magnesium acetate, 1 mM dithiothreitol (PH = 7.9)) were purchased from New England Biolabs (Ipswich, MA, USA). The ThT and sodium cefotaxime were obtained from Shanghai Aladdin Biochemical Technology Co., Ltd. (Shanghai, China). Various enzymes including (Bgl II, Not I, Hind III, Sac I, EcoR I) were purchased from Takara Biotechnology. Exonuclease III (Exo III) and Endonuclease IV (Endo IV) were obtained from Beyotime Biotechnology. Water was purified by Milli-Q filtration system, and all reagents were analytical grade and used as received. The DNA oligonucleotides used in this experiment were synthesized and purified by Sangon Biotech (Shanghai, China), and the details of the DNA oligonucleotide sequences are shown in (Table 1). In Table 1, probes HP-T, HP-C, and HP-G denote hairpin structures in which the nucleobase opposite the AP site is T, C and G, respectively. Meanwhile, the probes labeled A-2, A-3, A-7, and A-9 indicate that the AP site is located 2, 3, 7, and 9 nucleotides away from the 5′ end of the HP, respectively.

### 2.3. Standard Procedure for APE1 Determination

First, the synthesized H1, H2, and HP probe were incubated at 95 °C for 5 min, and then slowly cooled to room temperature to form a stable hairpin structure. For standard APE1 determination, a 20 µL mixture containing HP (2 µL, 1 µM), H1 (2 µL, 2.5 µM), H2 (2 µL, 2.5 µM), 10× NEBuffer 4 (2 µL), ThT (2 µL, 50 µM) and varying concentrations of APE1 were incubated at 37 °C for 30 min. The samples without APE1 treatment were used as the control sample. After incubation, the reaction system was diluted with 50 µL of water, and the fluorescence intensity was immediately measured using a photoluminescence spectrometer (FLSP920, Edinburgh Instruments, Livingston, UK). Under excitation at 445 nm, the emission spectrum was acquired over the wavelength range of 455–580 nm, and the fluorescence intensity recorded at 485 nm was used for subsequent quantitative analysis.

### 2.4. Specificity of the Proposed APE1 Assay

In specificity evaluation, 1.0 × 10^−5^ U/mL of inactive APE1 and different enzymes were selected as the potential interfering enzymes compared with 1.0 × 10^−5^ U/mL APE1. The activity of APE1 and other enzymes were detected using the same standard procedure stated above. 1.0 × 10^−5^ U/mL is the final concentration of the enzyme in the solution.

### 2.5. Inhibition of the APE1 Detection Assay

In the APE1 inhibitor assay, two model compounds, 7-nitroindole-2-carboxylic acid (NCA) and cefotaxime sodium, were used in this work to investigate the effects of inhibitors on the APE1-catalyzed DNA cleavage. The inhibition studies were carried out with similar procedures to those used for the standard APE1 determination assay stated above except that the inhibitor with varied concentrations were introduced into the reaction system.

### 2.6. Polyacrylamide Gel Electrophoresis (PAGE) Analysis

Different DNA solutions (10 µL per tube) mixed with 2 µL 6× loading buffer, which were then injected into 24% polyacrylamide hydrogel in 1× Tris-Borate-EDTA (TBE) buffer. Electrophoresis was performed at a constant voltage of 180 V for 90 min. Subsequently, the gel was stained with 4S Red Plus and visualized using a Syngene GBox imaging system (Syngene, Cambridge, UK).

### 2.7. Statistical Analysis

All quantitative experiments were performed in at least three independent replicates (*n* ≥ 3), with data presented as the mean ± standard deviation (SD). Statistical significance between two groups was determined using Student’s *t* test. A *p*-value of less than 0.05 was considered statistically significant (* *p* < 0.05, ** *p* < 0.01, *** *p* < 0.001, **** *p* < 0.0001). All statistical analyses were performed using GraphPad Prism software, version 8.0.

## 3. Results and Discussions

### 3.1. The Principle of Proposed Method for APE1 Activity Detection

**Scheme 1:**
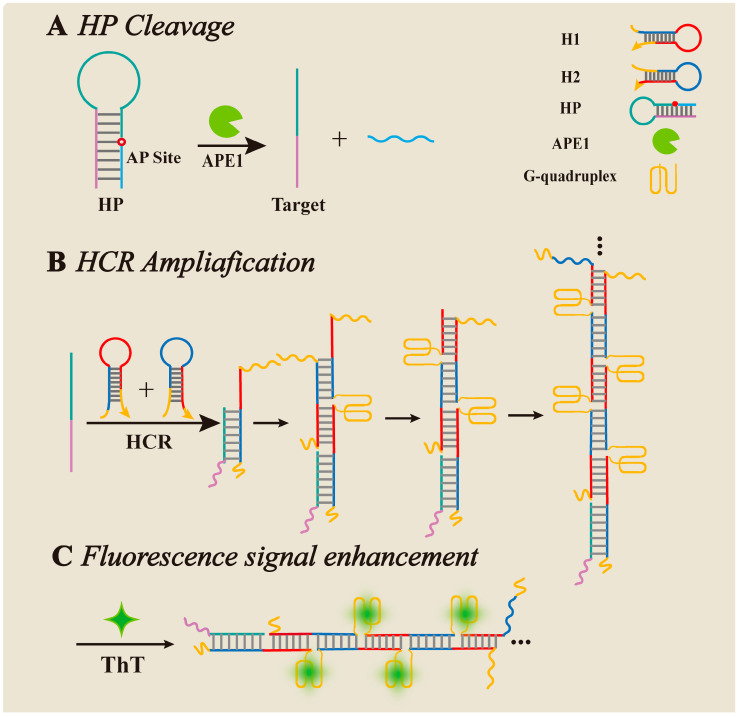
Schematic illustration of the ultrasensitive fluorescence biosensing platform for APE1 activity detection based on HCR and G-quadruplex amplification. The mechanism involves the following steps: (1) HP Cleavage: The HP contains AP site, which is specifically recognized and cleaved by the target enzyme APE1. This cleavage event releases a single-stranded HCR initiator sequence (green). (2) HCR Amplification: The liberated initiator (green) sequentially opens two stable hairpin probes, H1 and H2, via toehold-mediated strand displacement. This triggers a cascade of hybridization events, self-assembling long nicked double-stranded DNA polymers with numerous protruding G-quadruplex sequences (yellow). (3) Fluorescence signal enhancement: The fluorescent dye, ThT (green star), binds to the assembled G-quadruplex structures, resulting in a significant enhancement of fluorescence intensity. This light-up signal is directly proportional to the concentration of APE1 activity.

The working principle of the proposed APE1 activity analysis assay is shown in Scheme 1. As we know, APE1 can catalyze the hydrolysis of the phosphodiester backbone at the AP site. To analyze the activity of APE1, the HP probe containing HCR initiator was first designed. The designed HP contains four regions, the stem complimentary sequence (purple part), HCR initiator sequence (green part), blocking sequence (blue part) and AP site. In addition, two species of stem–loop DNA hairpins (H1 and H2) caged with a G-quadruplex sequence (yellow part) are rationally designed as the HCR fuels, which can stably coexist in the solution in the absence of the HCR initiator [17]. In the presence of APE1, APE1 would immediately recognize and cleave the AP site on blocked trigger, consequently release the blocking sequence and liberate the HCR initiator. In this regard, upon the addition of H1 and H2, the liberated HCR initiator will pair with the sticky end of H1 and open its hairpin through an unbiased strand-displacement reaction. Subsequently, the newly released sticky end of H1 will further hybridize with the sticky end of H2 to open the H2 hairpin and further expose a new single stranded sequence, which is identical to the HCR initiator. Therefore, a new cycle of H1 and H2 hybridization is consequently initiated. In this manner, each liberated HCR initiator triggers a hybridization chain reaction by sequentially and alternately binding to hairpin H1 and then hairpin H2, leading to the self-assembly of a long DNA polymer. Meanwhile, the G-quadruplex sequence encaged at the ends of H1 and H2 are completely exposed. In the presence of ThT, the two split G-quadruplex subunits drawn together to form G-quadruplex/ThT duplex and produce a significant fluorescence signal enhancement. In contrast, if APE1 is absent, the blocked HCR initiator was unable to trigger HCR and resulting signal enhancement. Therefore, in this design, APE1 activities are quantitatively reflected by the fluorescence intensity at 485 nm.

### 3.2. Feasibility of the Proposed APE1 Assay

**Figure 1:**
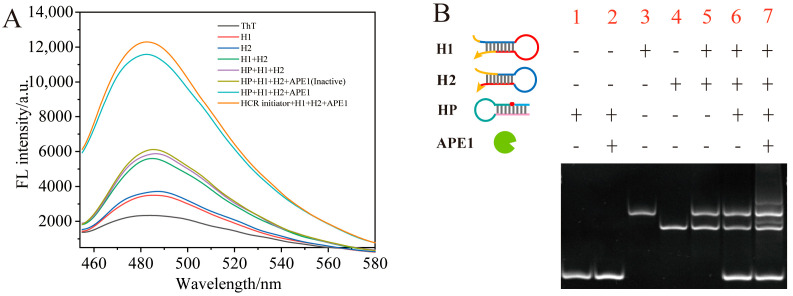
The feasibility of the proposed method. (**A**) Fluorescence spectrum under varying experimental conditions; (**B**) PAGE analysis of different samples: Trigger (lane 1); HP + APE1 (lane 2); H1 (lane 3); H2 (lane 4); H1 + H2 (lane 5); HP + H1 + H2 (lane 6); HP + H1 + H2 + APE1 (lane 7).

To verify the feasibility of this proposed method, the fluorescence emission spectrums of different samples were first recorded. As observed in Figure 1A, ThT, H1 and H2 represent negligible fluorescence signal, and only weak fluorescence intensity was observed when HP added into the H1 + H2 system. In contrast, a significant increase in fluorescence intensity at 485 nm was detected in the presence of APE1, as the enzyme cleaved the AP site, released the initiator, and triggered HCR-based signal amplification. However, when inactivated APE1 was introduced, the emission spectrum remained comparable to the blank sample, confirming that APE1-mediated cleavage of the AP site is essential for signal generation. In addition, a synthetic HCR initiator was included as a positive control. It produced a stronger fluorescence signal than the APE1-dependent reaction group, as expected, since the direct initiator bypasses the enzymatic cleavage step. This result confirms both the high efficiency of the HCR system and the specific detection of APE1 activity through cleavage-dependent amplification. In addition, the polyacrylamide gel electrophoresis (PAGE) was performed to validate the assay mechanism. As depicted in Figure 1B, faint smeared bands were observed in lanes 5 and 6 (absence of APE1), whereas a much brighter high-molecular-weight smear band can be observed in the presence of all reagents (lane 7), demonstrating that HCR amplification occurs only upon APE1-dependent AP site cleavage. Collectively, these results confirm the feasibility of the proposed assay.

### 3.3. Optimization of Experimental Parameters

**Figure 2:**
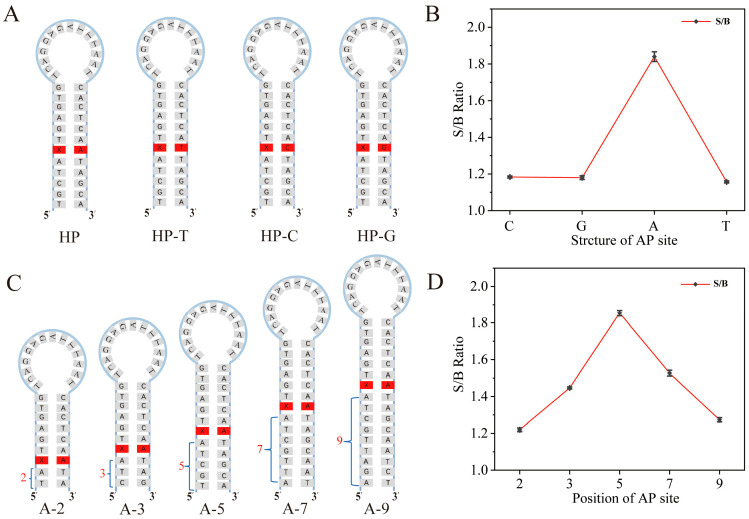
Optimization of experimental conditions. (**A**) Schematic representation of HP structures containing different AP site configurations (AAT, ATT, ACT, AGT). (**B**) Corresponding S/B ratios for APE1 activity with different AP site structures. (**C**) Schematic illustration of AP site positional variants (2–9 bases from 5′ end). (**D**) S/B ratios for APE1 activity at different AP site positions (*n* = 3).

To achieve the optimal analytical performance, related experimental parameters were systematically optimized. Since the structure and position of the AP site in HP significantly influence APE1’s hydrolysis activity, we first investigated how different AP site configurations affect enzymatic incision efficiency and detection sensitivity. Firstly, by keeping the sequence of AP site, the complementary sequence with four different base combinations (AAT, ATT, ACT and AGT) were tested. As shown in Figure 2A, the AAT combination yielded the highest signal-to-blank (S/B) ratio and was therefore selected for subsequent optimizations. Next, the optimal positioning of the AP site was examined. Figure 2B reveals that the S/B ratio increased markedly as the AP site was shifted from 2 to 5 bases from the 5′ end. However, further extension to 9 bases resulted in diminished performance. This trend can be attributed to two factors: (1) when the AP site is too close to the 5′ terminus, APE1 cleavage efficiency is suboptimal; (2) while moderate distancing (5 bases) enhances enzymatic activity, excessive separation may hinder the dissociation of the cleaved fragment from the HCR initiator, thereby impeding subsequent HCR amplification. Based on these findings, the AP site positioned 5 bases from the 5′ end was selected for all further experiments. Additionally, other critical parameters including incubation time, HP concentration, and H1/H2 concentrations were meticulously optimized (see Supplementary Information, Figures S1–S3) to ensure robust detection performance.

### 3.4. Analytical Performance of the Proposed APE1 Assay

**Figure 3:**
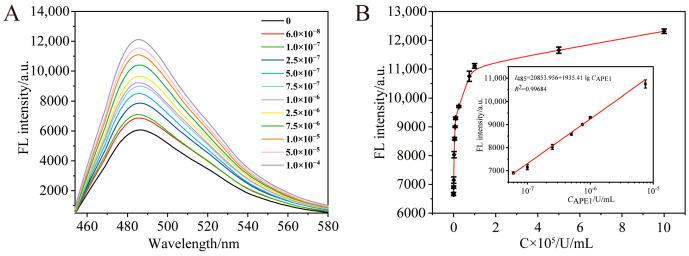
Detection performance analysis. (**A**) Fluorescence emission spectrum of the system in response to varying APE1 concentration (0, 6 × 10^−8^, 1 × 10^−7^, 2.5 × 10^−7^, 5 × 10^−7^, 7.5 × 10^−7^, 1 × 10^−6^, 2.5 × 10^−6^, 7.5 × 10^−6^, 1 × 10^−5^, 5 × 10^−5^ and 1 × 10^−4^ U/mL); (**B**) corresponding fluorescence intensity and linear relationship between fluorescence intensity at 485 nm and the logarithmic concentration of APE1 (*n* = 3).

Under optimal conditions, the detection performance of the proposed assay for APE1 was evaluated. Different concentration of APE1 was added into the proposed system and tested using a fluorescence spectrophotometer. As shown in Figure 3A, the fluorescence intensity at 485 nm increases progressively as the APE1 concentration increases from 0 to 1.0 × 10^−4^ U/mL. Figure 3B depicts the corresponding fluorescence intensity and a good linear correlation between fluorescence intensity and the logarithm of APE1 concentration can be observed in the range from 6.0 × 10^−8^ to 7.5 × 10^−6^ U/mL. The correlation equation is *I*_485_ = 20,853.956 + 1935.41 lg *C*_APE1_ (U/mL, *R*^2^ = 0.99684), and the corresponding detection limit is calculated to be 1.0 × 10^−8^ U/mL based on the 3 *σ*/*k* theory. As far as we know, the recently developed fluorometric and electrochemical methods coupled with either functional nanomaterials or DNA probes are the most popular and sensitive assays for APE1 (Table 2). The detection limits of APE1 using these protocols generally fall in the range of 1 × 10^−6^–1 × 10^−4^ U/mL. Notably, the sensitivity of the proposed assay is at least two orders of magnitude higher than that of most existing APE1 assays. This remarkably high sensitivity is largely attributed to the highly efficient signal amplification provided by HCR. To verify this, only H1 instead of H1/H2 is added as the fuel; therefore, each liberated HCR initiator will only capture one H1 hairpin and the hybridization reaction will be stopped. In such case, as shown in Figure S4, the detection limit of APE1 without HCR is only around 5 × 10^−5^ U/mL. These results clearly suggest that the efficient HCR amplification makes the detection sensitivity two orders of magnitude higher. To the best of our knowledge, the detection limit of APE1 (1.0 × 10^−8^ U/mL) by using the proposed method is the lowest obtained thus far for APE1 analysis.

### 3.5. Specificity, Repeatability, and Stability of the Proposed APE1 Assay

**Figure 4:**
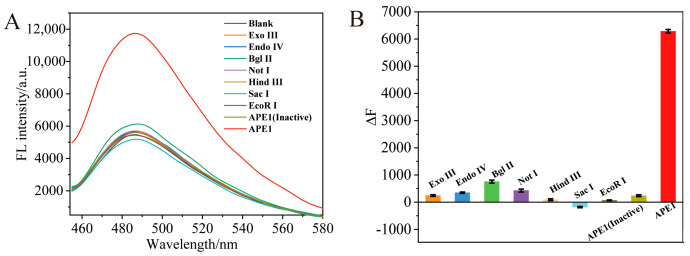
Specific analysis. (**A**) Fluorescence emission spectrum of the assay in response to various interferents and APE1. (**B**) Corresponding fluorescence intensities at 485 nm for each enzyme compared to active APE1 (*n *= 3).

Specificity represents a crucial performance metric for analytical methods. To evaluate the specificity of our assay, we challenged the system with various potential interferents, including Exo III, Endo IV, BgI II, Not I, Hind III, Sac I, EcoR I and inactivated APE1. As demonstrated in Figure 4, only active APE1 generated significant fluorescence enhancement, while all control enzymes produced comparable signals to the Blank sample. This pronounced signal discrimination confirms the method’s exceptional specificity for APE1, with no observable cross-reactivity toward other DNA-modifying enzymes.

In addition to sensitivity and specificity, we rigorously evaluated the repeatability and stability of this bioassay. For repeatability testing, intraday measurements of APE1 at three concentrations (1.0 × 10^−7^, 1.0 × 10^−6^ and 1.0 × 10^−5^ U/mL) were performed in three independent experiments. The resulting relative standard deviation (RSD) values were consistently below 5% (Figure S5), confirming their excellent repeatability. To evaluate stability, APE1 samples (1.0 × 10^−5^ U/mL) were analyzed daily over seven days of storage at 4 °C. As demonstrated in Figure S6, the minimal signal variation (RSD = 3.29%) establishes the method’s stability for both immediate measurements and short-term (7-day) sample storage under refrigeration.

### 3.6. Application of the Proposed Assay in Actual Samples

**Figure 5:**
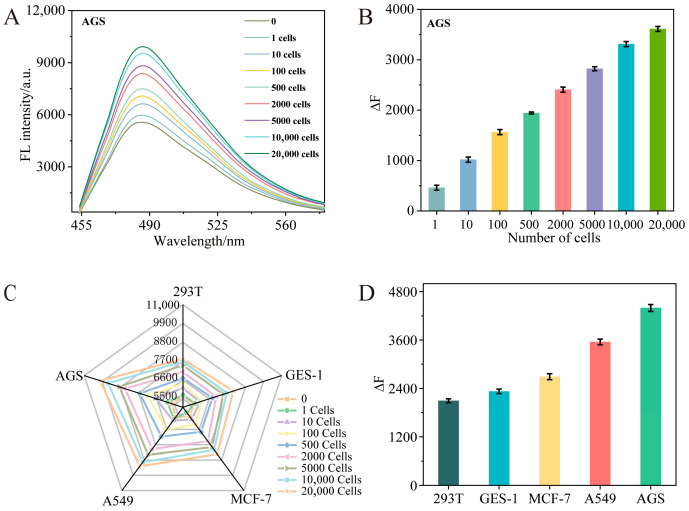
APE1 activity in biological samples. (**A**) Fluorescence emission spectra of AGS cell lysates across a cellular concentration range (0–20,000 cells). (**B**) Corresponding fluorescence intensities at 485 nm for AGS cell lysates. (**C**) Radar chart comparing relative APE1 activity profiles across five cell types. (**D**) Statistical comparison of APE1 activity levels among the five cell lines at 20,000 cells per sample (*n *= 3).

Furthermore, a practical bioassay should be applicable for target analysis in complex biological samples. To evaluate the robustness of the proposed method, we further investigate its analytical performance for APE1 determination in cell lysates. Initial validation employed gastric cancer (AGS) cell lysates with cell counts ranging from 0 to 20,000. As shown in Figure 5A,B, the fluorescence intensity at 485 nm increased proportionally with cell number, indicating cell-number-dependent AP site cleavage and subsequent G-quadruplex formation. We further validated the method’s generalizability across multiple cell lines, including breast cancer (MCF-7), lung cancer (A549), embryonic kidney (293T), and normal gastric epithelium (GES-1) cells (Figure S7). Notably, cancer cell lines exhibited significantly higher APE1 activity than normal cells at equivalent cell numbers (Figure 5C,D). Remarkably, the assay demonstrated sufficient sensitivity for single-cell APE1 activity detection, highlighting its potential for advanced biomedical applications. Furthermore, the APE1 mRNA expression in the same panel of cell lines was evaluated by conventional RT-qPCR. As shown in Figure S8, elevated APE1 transcript levels were observed in cancer cells (MCF-7, A549, and AGS) compared to normal controls (GES-1 and 293T), consistent with our results. However, it is well-established that mRNA abundance may not directly correlate with enzymatic activity due to post-transcriptional and post-translational regulatory mechanisms. This distinction is clearly illustrated by our results: for instance, AGS cells exhibited lower APE1 mRNA levels than A549 cells, yet demonstrated significant APE1 enzymatic activity in our proposed assay. While RT-qPCR remains invaluable for gene expression profiling, our method provides a unique and complementary approach by directly quantifying APE1’s functional endonuclease activity, offering deeper insight into its biological role in DNA repair. Moreover, the biological variability in APE1 activity detected across different cell lines underscores this assay’s potential for functional phenotyping. The consistent measurement of these differences confirms the method’s robustness in complex biological matrices and its sensitivity to physiologically relevant changes, which is essential for future analysis of clinical samples.

To validate the assay’s accuracy, we performed standard addition recovery tests using cell lysate from two prototypical cell lines: GES-1 (10,000 cells, representing a normal cell line) and AGS (500 cells, representing a cancer cell line). The endogenous APE1 activities were determined to be 2.00 × 10^−7^ U/mL (GES-1) and 1.80 × 10^−7^ U/mL (AGS), respectively (Table 3). Known quantities of standard APE1 (1.00 × 10^−7^, 3.00 × 10^−7^ and 5.00 × 10^−7^ U/mL) were spiked into each lysate and the additional concentrations were tested to be 1.00 × 10^−7^, 2.91 × 10^−7^ and 5.02 × 10^−7^ U/mL for GES-1 and 9.89 × 10^−8^, 3.02 × 10^−7^ and 4.98 × 10^−7^ U/mL for AGS. These results demonstrate the excellent accuracy (97.29–100.89% recovery) and matrix-independent reliability of the assay, supporting its suitability for APE1 quantification in complex biological samples.

### 3.7. APE1 Inhibition Assay

**Figure 6:**
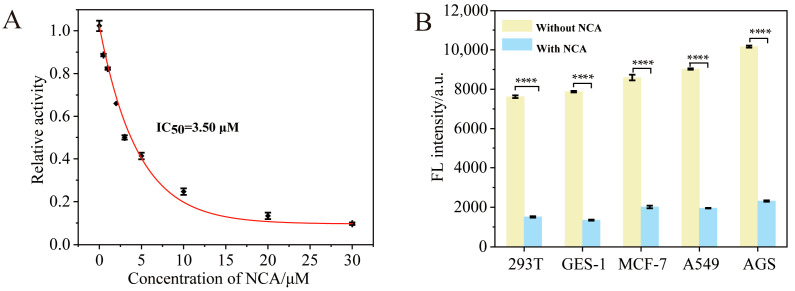
Inhibitor analysis. (**A**) Dose-dependent inhibition of APE1 activity by NCA treatment. (**B**) Comparative fluorescence intensity analysis of APE1 activity in five cell lines following NCA treatment (*n *= 3). Statistical significance was determined by an unpaired, two-tailed Student’s *t*-test; **** *p* < 0.0001 versus the control group.

It is well-established that APE1 serves as a promising therapeutic target in oncology. As the indispensable endonuclease in the base BER pathway, APE1 overexpression is frequently associated with enhanced DNA repair capacity, tumor progression, and acquired resistance to DNA-damaging chemotherapeutic agents such as temozolomide and cisplatin. Consequently, the development of effective APE1 inhibitors represents a compelling strategy for sensitizing cancer cells to conventional treatments by disrupting this critical repair mechanism and tipping the cellular balance toward apoptosis. [16]. In the present study, we evaluated our assay’s capability for inhibitor screening using two known APE1 inhibitors: NCA and cefotaxime sodium. With APE1 fixed at 1 × 10^−5^ U/mL, dose-dependent inhibition was observed for both compounds. As shown in Figure 6, increasing NCA concentrations (0–30 μM) produced a progressive decrease in fluorescence intensity at 485 nm, demonstrating effective enzymatic inhibition. Parallel experiments with cefotaxime sodium (0–100 μM) showed a similar dose–response behavior (Figure S9). Quantitative analysis revealed IC_50_ values of ~3.50 μM for NCA and ~75 μM for cefotaxime sodium. Notably, both inhibitors maintained efficacy in cell lysate matrices, demonstrating our assay’s utility for high-throughput screening of APE1 inhibitors in both simplified systems and biologically relevant environments. These results highlight the potential of this enzyme-free sensing platform to facilitate the discovery and characterization of novel APE1 inhibitors, which may contribute to the development of adjuvant therapies aimed at overcoming chemoresistance in cancer.

### 3.8. Evolution Towards Point-of-Care Diagnostics

While the current homogeneous format of the assay lends itself well to high-throughput screening applications, its dependence on fluorescence readout limits its utility in point-of-care settings. To broaden its diagnostic applicability, future work could focus on integrating a colorimetric readout strategy. For instance, engineering the HCR products to form G-quadruplex structures would allow subsequent binding with hemin to create DNAzyme mimics with peroxidase-like activity. These DNAzymes can catalyze the oxidation of substrates such as TMB (3,3′,5,5′-Tetramethylbenzidine), producing a visible color change detectable by the naked eye or simple portable devices. This modification would enable instrument-free visual detection, significantly enhancing the platform’s suitability for resource-limited environments. Furthermore, the inherent stability of synthetic DNA components ensures long-term viability, providing excellent prospects for practical deployment.

## 4. Conclusions

In summary, we have developed an ultra-sensitive and selective biosensor for APE1 activity detection based on a structurally optimized HP probe combined with HCR and ThT-assisted signal amplification. This assay offers multiple advantages. Firstly, strategic optimization of the HP probe’s AP site structure and localization markedly improved APE1 enzymatic activity, resulting in optimal signal amplification efficiency. Secondly, the highly efficient APE1-mediated cleavage coupled with subsequent HCR creates a robust signal augmentation effect. Thirdly, leveraging the pronounced fluorescence enhancement from ThT-G-quadruplex interactions enabled the development of a highly reliable, sensitive, and selective APE1 detection platform. This biosensor has been successfully applied to measure APE1 activity in complex biological samples and screen potential APE1 inhibitors, demonstrating significant promise for cancer diagnosis and APE1-targeted drug development.

## Figures and Tables

**Table 1:** Sequences of oligonucleotides used in the present work ^a^.

Name	Sequence (5′-3′)
H1	TGGGTTTAATCTCCTGACACTCATGTTAAGAGGTTGAGTGTCAGGGTAGGGCGGGA
H2	AGGGCGGGTGGGTTGAGTGTCAGGAGATTAATGACACTCAACCTCTTAACTGGGA
HCR initiator	TGAGTGTCAGGAGATTAATCACTCAATAGCA
HP	TGCTAXTGAGTGTCAGGAGATTAATCACTCAATAGCA
HP-T	TGCTAXTGAGTGTCAGGAGATTAATCACTCATTAGCA
HP-C	TGCTAXTGAGTGTCAGGAGATTAATCACTCACTAGCA
HP-G	TGCTAXTGAGTGTCAGGAGATTAATCACTCAGTAGCA
A-2	TAXTGAGTGTCAGGAGATTAATCACTCAATA
A-3	CTAXTGAGTGTCAGGAGATTAATCACTCAATAG
A-7	ATTGCTAXTGAGTGTCAGGAGATTAATCACTCAATAGCAAT
A-9	AGATTGCTAXTGAGTGTCAGGAGATTAATCACTCAATAGCAATCT

^a^ Underline sequences indicate complementary regions of the probes to form hairpin structure. X represents AP sites.

**Table 2:** Comparison of the analytical performance of various methods for APE1 detection.

Strategy	Detection Mode	Linear Range (U/mL)	LOD (U/mL)	Ref.
HRCA	Fluorescence	0.0001–0.2	1.0 × 10^−4^	[15]
TdT/Endo IV	Fluorescence	2.0 × 10^−6^–0.004	1.7 × 10^−6^	[16]
CHA	Fluorescence	5.0 × 10^−6^–30.0	4.78 × 10^−6^	[22]
DNAzymes	Colorimetry	2.5–22.5	1.8	[23]
AuCu/GO	SERS	0.002–20	0.001	[24]
CHA-HCR	Electrochemical	0.001–1	0.001	[25]
CHA	Electrochemistry	0.01–4	0.005	[26]
CRISPR/Cas12a	Fluorescence	0.0025–25.0	2.5 × 10^−4^	[27]
Ni-AuNCs	Electrochemical	8.6 × 10^−6^–8.6 × 10^−2^	3.34 × 10^−6^	[28]
DEP-Cas-APE	Fluorescence	0.0001–1.0	7.66 × 10^−5^	[29]
HCR/CHA	Fluorescence	0.0001–1.0	7.80 × 10^−5^	[30]
RCA/G-quadruplex	Fluorescence	2.0 × 10^−6^–10	1.52 × 10^−6^	[31]
DNA Walker/AuNPs	Fluorescence	0.01–100	5.54 × 10^−3^	[9]
HCR	Fluorescence	6.0 × 10^−8^–7.5 × 10^−6^	1.0 × 10^−8^	This work

**Table 3:** Recovery results of the determination of APE1 in cell lysates (*n* = 5).

Sample	Content/U/mL	Added/U/mL	Measured/U/mL	Found/U/mL	Recovery/%	RSD/%
GES-1	2.00 × 10^−7^	1.00 × 10^−7^	3.00 × 10^−7^	1.00 × 10^−7^	100.76	3.56
		3.00 × 10^−7^	4.91 × 10^−7^	2.91 × 10^−7^	97.29	2.32
		5.00 × 10^−7^	7.02 × 10^−7^	5.02 × 10^−7^	99.57	3.03
AGS	1.80 × 10^−7^	1.00 × 10^−7^	2.79 × 10^−7^	9.89 × 10^−8^	98.88	2.94
		3.00 × 10^−7^	4.82 × 10^−7^	3.02 × 10^−7^	100.89	3.04
		5.00 × 10^−7^	6.78 × 10^−7^	4.98 × 10^−7^	99.64	3.23

## Data Availability

The original contributions presented in this study are included in the article. Further inquiries can be directed to the corresponding author.

## Supplementary Materials

The following supporting information can be downloaded at: https://www.mdpi.com/article/10.3390/biom15091275/s1, Figures S1–S3: Optimization of the experimental conditions of the system. Figure S4: Effects of HCR. Figures S5 and S6: Repeatability and stability analysis of the biosensor. Figure S7: Application of the proposed assay in actual samples. Figure S8: APE1 mRNA expression level. Figure S9: APE1 inhibition assay.
