# Supplementary material for: Label-Free and Ultrasensitive APE1 Detection Based on Hybridization Chain Reaction Combined with G-Quadruplex
Source: Biomolecules. 2025 Sep 3;15(9):1275. doi: 10.3390/biom15091275 (PMC12467234; doi:10.3390/biom15091275)
Supplement: Supplementary file 1 [file biomolecules-15-01275-s001.zip › biomolecules-3809493-supplementary.pdf]

Supporting Information

Label-Free and Ultrasensitive APE1 Detection Based on Hybridization Chain Reaction Combined with G-Quadruplex

Yarong Zhang 1,†, Hongyan Ma 1,†, Zhenyao Gao 2, Miao Li 1, Fan Yang 1, Lingbo Sun 2 and Yuecheng Zhang 1,*

1 Key Laboratory of Analytical Technology and Detection of Yan'an, College of Chemistry and Chemical Engineering, Yan'an University, Yan'an 716000, China

2 Medical College of Yan'an University, Yan'an University, Yan'an 716000, China

* Correspondence: yuechengzhang@yau.edu.cn

† These authors contributed equally to this work.

1. Optimization of the experimental conditions of the system

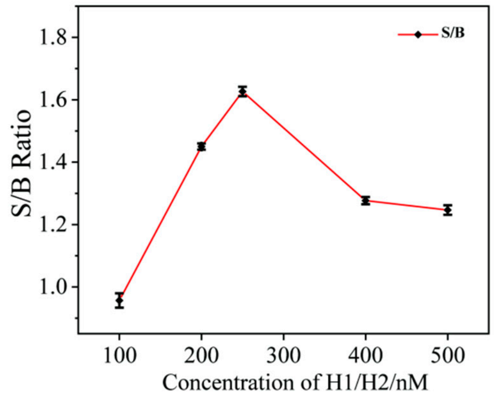

Figure S1. Optimization the concentration of H1 and H2 (Error bars: n=3).

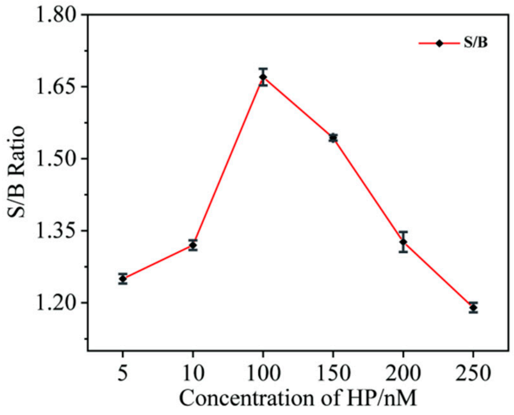

Figure S2. Optimization the concentration of HP (Error bars: n=3).

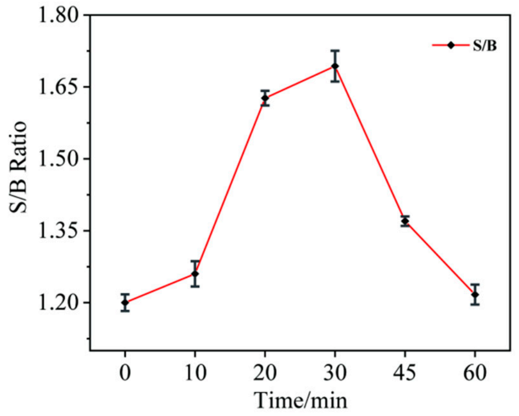

Figure S3. Optimization of the reaction time (Error bars: n=3).

2. Effects of HCR

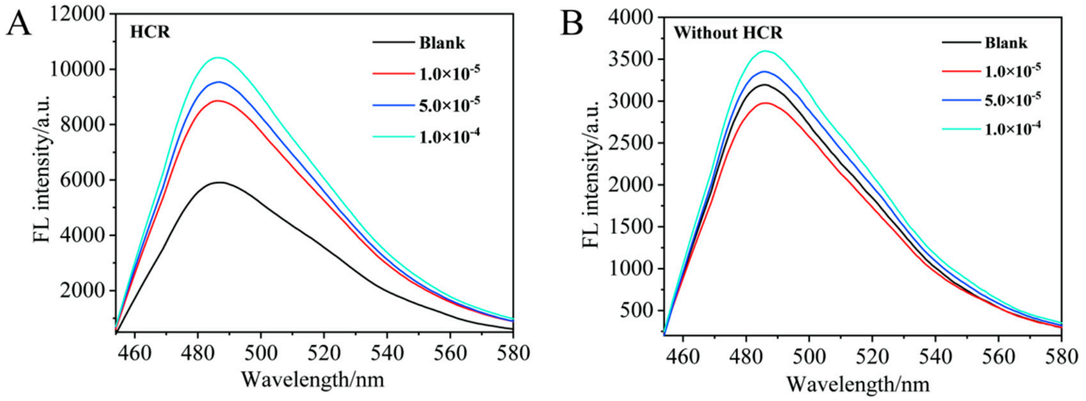

Figure S4. Effects of HCR. (A)Fluorescence spectra of the system with HCR amplification; (B) Fluorescence spectra of the system by only use H1 as the fuel. Concentration of APE1(1×10^{-5}、5×10^{-5}、1×10^{-4} U/mL)

3. Repeatability and stability analysis of the biosensor

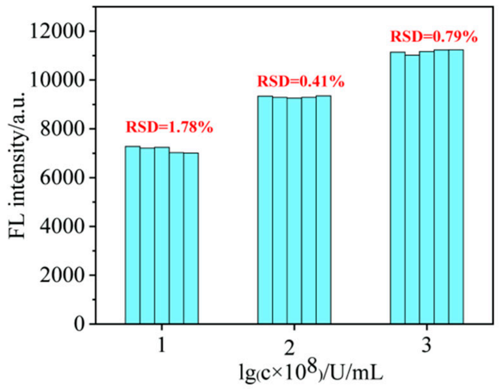

Figure S5. Repeatability of the proposed APE1 assay (APE1 concentration: 1×10^{-7}、1×10^{-6}、1×10^{-5} U/mL, Error bars: n=3).

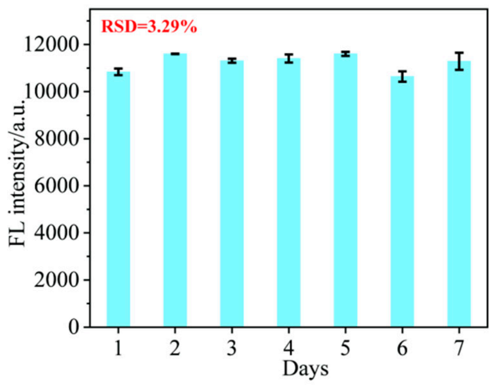

Figure S6. Stability of proposed APE1 assay (APE1 concentration: 1×10^{-5} U/mL, Error bars: n=3)

4. Application of the proposed assay in actual samples

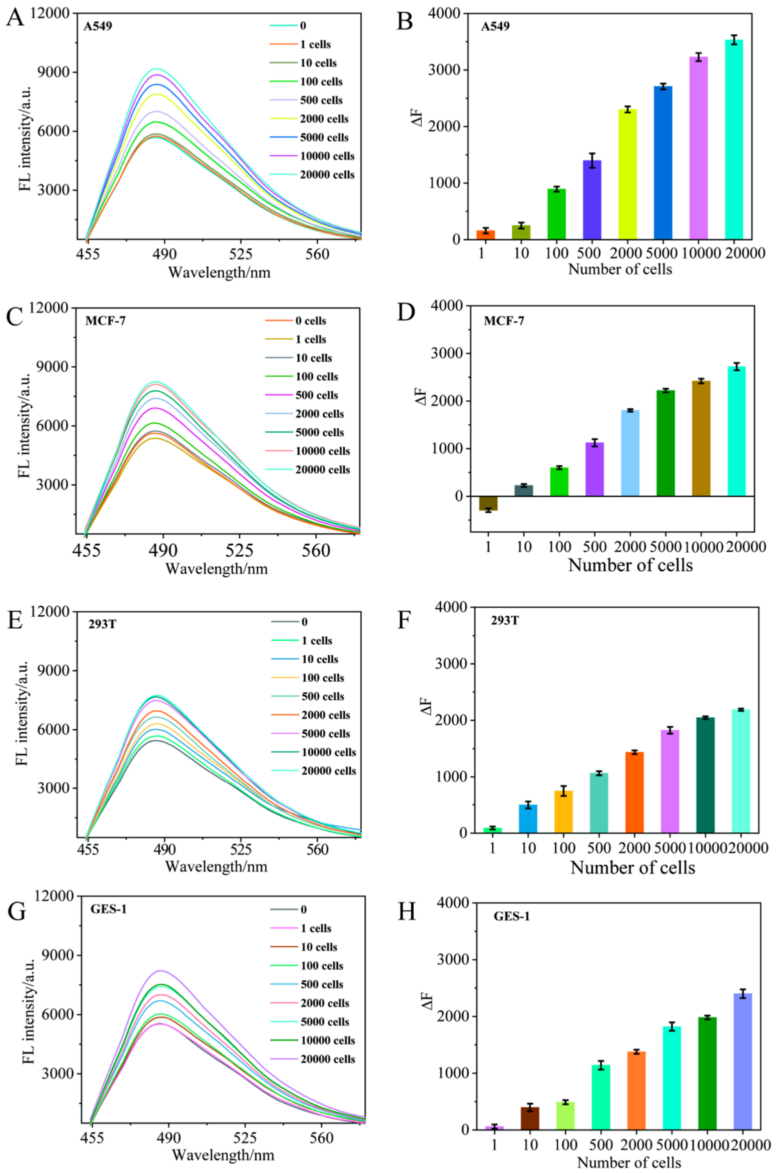

Figure S7. (A) Fluorescence spectra of A549 cell lysates (0–20,000 cells). (B) Corresponding

fluorescence intensity quantification for A549 cells. (C) Fluorescence spectra of MCF-7 cell lysates (0–20,000 cells). (D) Corresponding fluorescence intensity quantification for MCF-7 cells. (E) Fluorescence spectra of 293T cell lysates (0–20,000 cells). (F) Corresponding fluorescence intensity quantification for 293T cells. (G) Fluorescence spectra of GES-1 cell lysates (0–20,000 cells). (H) Corresponding fluorescence intensity quantification for GES-1 cells (Error bars: n=3).

5. APE1 mRNA expression level

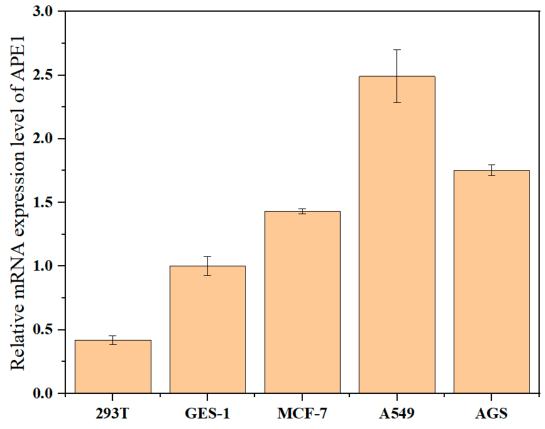

Figure S8. Relative mRNA expression level of APE1 (Error bars: n=3).

6. APE1 inhibition assay

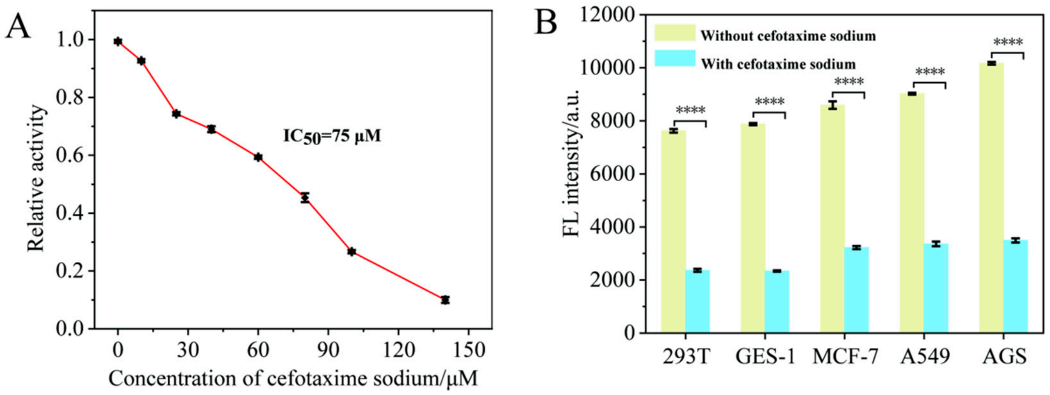

Figure S9. (A) Relative APE1 activity changes under varying concentrations of cefotaxime sodium (APE1 concentration: 1×10^{-5} U/mL). (B) Fluorescence intensity comparison of five cell lysates with/without cefotaxime sodium treatment (Error bars: n=3).
